# Deep learning-enhanced signal detection for communication systems

**DOI:** 10.1371/journal.pone.0324916

**Published:** 2025-05-27

**Authors:** Yang Liu, Peng Liu, Yu Shi, Xue Hao

**Affiliations:** Department of Communication Electronic Countermeasure, Aviation University of Air Force, Changchun, China; Guangdong University of Petrochemical Technology, CHINA

## Abstract

Traditional communication signal detection heavily relies on manually designed features, making it difficult to fully characterize the essential characteristics of the signal, resulting in limited detection accuracy. Based on this, the study innovatively combines Multiple Input Multiple Output (MIMO) with orthogonal frequency division multiplexing technology to construct a data-driven detection system. The system adopts a Multi-DNN method with a dual-DNN cascade structure and mixed activation function design to optimize the channel estimation and signal detection coordination process of the MIMO part. At the same time, a DCNet decoder based on a convolutional neural network batch normalization mechanism is designed to suppress inter-subcarrier interference in OFDM systems effectively. The results showed that on the simulation training set, the accuracy of the research model was 93.8%, the symbol error rate was 17.6%, the throughput was 81.3%, and the modulation error rate was 0.004%. On the simulation test set, its accuracy, symbol error rate, throughput, and modulation error rate were 90.7%, 18.1%, 81.2%, and 0.006%. In both 2.4 GHz and 5 GHz WiFi signals, the signal detection accuracy of the research model reached 91.5% and 91.6%, with false detection rates of 1.9% and 1.5%, and missed detection rates of 1.6% and 4.2%. In resource consumption assessment, the detection speed of this model reached 120 signals/s, with an average latency of 50 ms. The model loading time was only 2.4 s, and the CPU usage was as low as 25%, with moderate memory usage. Overall, the research model has achieved significant results in improving detection accuracy, optimizing real-time performance, and reducing resource consumption. It has broad application prospects in the field of communication signal detection.

## 1. Introduction

The advancement of information technology has made communication systems indispensable in people’s daily lives and work, becoming a bridge connecting the world. Whether it is instant communication between people or emerging technologies including the Internet of Things (IoT), big data, and cloud computing, efficient and stable communication systems are indispensable as support [1]. However, the continuous development of communication technology has made the complexity of the communication environment increasingly prominent. The amount of communication data has also shown explosive growth. This is undoubtedly a huge challenge for traditional Communication Signal Detection (CSD) methods. Traditional CSD methods are mostly based on manually designed features and fixed algorithm models. These methods and models have played an important role in simple and stable communication environments in the past. However, in the face of the current complex and ever-changing communication environment, such as multi-path propagation, fading, interference, and other complex factors, traditional methods seem inadequate. In addition, with the advent of the big data era, the amount of communication data has increased sharply. Traditional signal detection methods still face problems such as low efficiency when processing large-scale data. Based on this, exploring new CSD technologies to improve detection precision and efficiency has become a vital research topic in communication in recent years.

Jiang X et al. designed a novel signal detection algorithm using stochastic resonance technology to reduce interference between signals. This algorithm achieved effective detection under low Signal-to-Noise Ratio (SNR) signal conditions and could improve the performance of the entire satellite communication system [2]. Basak et al. used the frequency characteristics of the transmitted signal for RF-based drone signal detection to achieve drone localization. Compared with the goodness of fit spectrum sensing, the YOLO framework provided better detection performance for synchronous multi-signal scenes [3]. Emir A et al. proposed a Deep Learning-assisted Multi-user Detection (DeepMuD) technique. This technology was applied in uplink Non-orthogonal Multiple Access (NOMA) systems to enhance large-scale machine type communication. In this system, multi-user detection was achieved through a long short-term memory network based on offline training. The proposed DeepMuD significantly improved the error performance of uplink NOMA and outperformed traditional detectors. In addition, DeepMuD also had flexible detection capabilities that could perform multi-user detection regardless of the number of devices. Therefore, it could provide services to any number of IoT devices without signal overhead, achieving unauthorized communication [4]. Ye et al. utilized signal samples received by environmental backscatter communication receivers to estimate a detection threshold close to optimal. The accuracy of the Symbol Error Rate (SER) and detection threshold derived by this method was higher. Its detector was more robust to amplitude imbalance than phase imbalance [5]. Li S et al. put forth a novel mixed-signal detection algorithm utilizing orthogonal time-frequency space modulation to provide reliable communication in high mobility scenarios. Compared with existing algorithms, this method could significantly improve system performance [6]. Joshi R et al. first combined deep learning with 3D integrated imaging for simultaneous underwater object detection and optical signal detection. The experimental results showed that the model provided better image reconstruction performance in degraded environments and had good detection accuracy [7]. Wu Y et al. proposed a signal detector based on a combination of Orthogonal Time-Frequency Space (OTFS) modulation and Index Modulation (IM) system. A linear equalizer based on Minimum Mean Square Error (MMSE) and its corresponding soft decision were added, as well as a Vector-Assisted Message Passing (VV-MP) detector and its related soft decision. The experimental results showed that the OTFS-IM system using these detectors could provide significant Bit Error Rate (BER) performance improvement compared to OTFS, Orthogonal Frequency Division Multiplexing (OFDM), and IM systems [8]. Q Tao et al. proposed an OTFS modulation scheme combining Intelligent Reflective Surface (IRS) to solve the problem of signal power drop caused by incoherent superposition in high mobility scenarios. They designed the OTFS frame structure and IRS phase shift to a achieve coherent combination of received signals. To address the issue of outdated channel state information in high mobility systems, a location-assisted IRS channel estimation strategy was proposed. In addition, to mitigate the adverse effects of fractional Doppler frequency shift, a channel estimation method based on delay and shifted Doppler domains was designed. A low-complexity Iterative Interference Cancellation (IIC) detector was proposed using a carefully designed OTFS frame structure and IRS phase offset. Additionally, the lower bound of its symbol error probability was analyzed. The experimental results showed that the proposed estimation method and IIC detector have been validated for their effectiveness and superiority [9].

Although previous research has achieved certain results, there is still room for improvement. The MIMO-OFDM CSD method, which combines data-driven Multiple Input Multiple Output (MIMO) and OFDM, has improved the automation and intelligence level of detection to a certain extent. However, this model usually contains a large number of parameters and complex network structures. It can easily increase the computational complexity of the model during training and inference, thereby affecting the real-time performance and energy efficiency of detection. Based on this, the study aims to construct a CSD system that combines high precision and high efficiency by integrating deep learning techniques. The specific objectives include: (1) designing a Multi-Task Deep Neural Network (Multi-DNN) module with a dual-DNN cascade structure to achieve joint optimization of channel estimation and signal detection in MIMO systems. (2) Constructing a Decoding Networks (DCNet) decoder based on deep feature extraction to address the sensitivity issue of sub-carrier interference in OFDM systems. (3) Establishing an end-to-end lightweight inference framework and building a high-precision and low-latency CSD system through the collaborative optimization of Multi-DNN and DCNet. The innovation of this model lies in the introduction of Multi-DNN to optimize the MIMO part and the use of DCNet to optimize the OFDM part. This dual-module collaborative optimization design provides a new technical path for solving the performance bottleneck of traditional methods in complex communication environments.

## 2. Methods and materials

### 2.1. MIMO-OFDM model based on dnn data-driven approach

**Fig 1:**
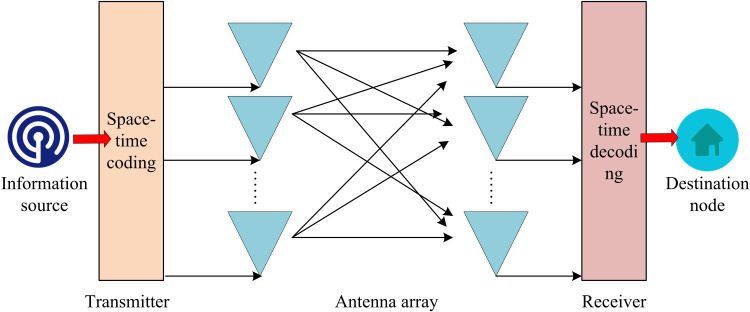
MIMO structure.

In modern wireless communication environments, multi-path effects and bandwidth efficiency are key issues that must be thought about during signal transmission. The impact of multi-path effects on wireless communication systems is mainly reflected in signal distortion and inter-symbol interference. The impact of frequency selective fading on wireless communication systems is mainly reflected in the decrease in communication quality and limited transmission rate. Bandwidth efficiency reflects the system’s ability to transmit data within a limited bandwidth. These issues will directly affect the performance, capacity, and user experience of wireless communication systems. Therefore, when designing and optimizing wireless communication systems, it is required to fully consider these factors and adopt proper measures to counteract them. MIMO-OFDM communication system is a wireless communication system that combines MIMO and OFDM technologies, which can effectively address the above-mentioned issues [10]. Among them, MIMO technology can resist multi-path fading to a certain extent. The structure of MIMO is shown in Fig 1.

In Fig 1, a source transmits signals through a transmitter, which contains multiple antennas. These antennas can simultaneously transmit multiple signal streams. Signal flow is transmitted through wireless channels, where there are multiple different propagation paths, resulting in multi-path propagation [11]. The receiving end also has multiple antennas, which are responsible for receiving multiple signal streams from the transmitting end. After processing, the received signal finally reaches the destination. It is precisely because multiple independent propagation paths are used to transmit signals that MIMO can still use other paths to transmit signals even if one path is interfered with or attenuated [12]. The relationship of MIMO is shown in formula (1).


$$y=\left(\begin{array}{cccc} {}*20ch_{11} & h_{12} & \cdots & h_{1N_{T}}\\ h_{21} & h_{22} & \cdots & h_{2N_{T}}\\ \cdots & \cdots & \cdots & \cdots \\ h_{N_{R}1} & h_{N_{R}2} & \cdots & h_{N_{R}N_{T}} \end{array}\right)s+v$$ (1)

**Fig 2:**
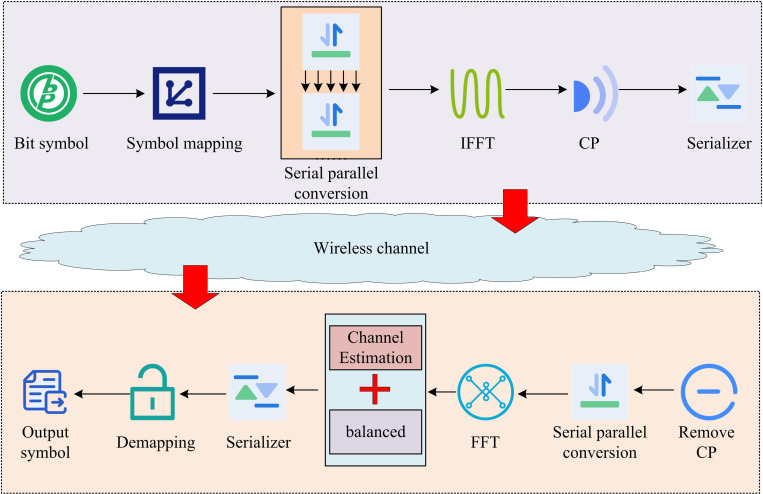
OFDM structure diagram.

In formula (1), y is the receiving vector. $h_{ij}$ is the element in the channel matrix. s is the transmitting vector. v is the noise vector. OFDM technology can divide the total bandwidth into several narrow sub-carriers, effectively resisting frequency selective fading, as shown in Fig 2.

In Fig 2, the structure includes a sender and a receiver. At the transmitting end, the original bit symbols are first mapped onto specific symbols, and then the serial data are converted into parallel data through Serial Parallel Conversion (SPC) for transmission on multiple sub-carriers [13]. Next, the Frequency-Domain Signal (FDS) is converted into a Time-Domain Signal (TDS) through inverse Fast Fourier Transform (FFT). A Cyclic Prefix (CP) is inserted to resist inter symbol interference caused by multi-path effects [14]. At the receiving end, CP is initially removed, and then the TDS is converted back to the FDS through SPC, followed by FFT to convert the signal back to the FDS [15]. The next step is to perform channel estimation and equalization to compensate for frequency selective fading of the channel. The final step is to restore the signal to its original bit symbols through Parallel-to-Serial (P/S) conversion and demapping, as shown in formula (2).


$$\left\{ \begin{array}{c} Y=H\times D+Z\\ \hat{D}=\mathrm{\backslash arg}\min_{\tilde{D}}∥Y-H\tilde{D}{∥}^{2} \end{array}\right.$$ (2)

In formula (2), Y, H, D, and Z are frequency domain representations of the received signal, channel matrix, transmitted signal, and noise after FFT. $\hat{D}$ and $\tilde{D}$ are the predicted values of the received and transmitted signals. By combining MIMO and OFDM technologies, a MIMO-OFDM system can be constructed to detect communication signals. However, MIMO-OFDM systems generally use MMSE detection in the detection of communication signals, which is usually based on known channel states and noise statistical characteristics for signal detection [16]. However, in practical applications, the channel state is often not fully known, and the statistical characteristics of noise may also vary over time [17]. In addition, MIMO-OFDM also has certain requirements for the communication environment. If the communication environment is too complex, MIMO-OFDM systems will still be affected by factors such as noise, interference, and channel variations [18]. To handle the aforementioned issues, the paper proposes the introduction of DNN to construct a data-driven CSD model. DNN has a multi-layer neural network structure, which enables it to have high nonlinear expression ability and learning ability of complex data patterns by stacking multiple hidden layers. Its core lies in forward propagation and backward propagation. The forward propagation calculation is shown in formula (3).


$$\{\begin{array}{c} {}*20lz^{l}=W^{l}a^{l-1}+b^{l}\\ a^{l}=\sigma (z^{l}) \end{array}$$ (3)

In formula (3), $z^{l}$ represents the linear output of the current layer. $W^{l}$ is the weight matrix for the l -th layer, which determines how the output from the previous layer affects the current layer’s output. $a^{l-1}$ is the output from the previous layer and $b^{l}$ is the bias term for the l -th layer. The number of layers is denoted by l. The activation function σ introduces nonlinearity, allowing the network to learn and model more complex functions. The specific expression of backpropagation is shown in formula (4).


$$\left\{ \begin{array}{c} \delta^{l}=\frac{\partial J}{\partial z^{l}}=(W^{l+1}{)}^{T}\delta^{l+1}⊙\sigma \\ W^{l}=W^{l}-\alpha \frac{\partial J}{\partial W^{l}}\\ b^{l}=b^{l}-\alpha \frac{\partial J}{\partial b^{l}} \end{array}\right.$$ (4)

**Fig 3:**
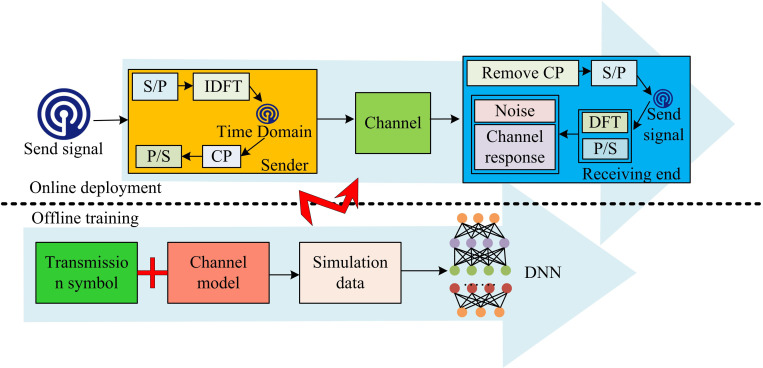
CSD model based on data-driven approach.

In formula (4), J is the loss function. α is the learning rate. δ is the error. Based on the linear expression and learning capabilities of DNN, MIMO-OFDM can automatically adjust network parameters and learn the mapping relationship between received and transmitted signals. It can adapt to different channel conditions and noise characteristics, achieving more accurate signal detection. The data-driven detection model based on DNN is shown in Fig 3.

Fig 3 shows the data-driven CSD model constructed after introducing DNN. At the transmitting end of the model, the original transmission symbols are first subjected to Serial-to-Parallel conversion (S/P). Then, the FDS is converted into a TDS through inverse Discrete Fourier Transform (DFT) [19]. Next, CP is inserted to resist inter-symbol interference caused by multi-path effects, and finally, the signal is transmitted into the channel through P/S conversion. At the receiving end, the CP in the received signal is first removed, and then the TDS is converted back to the FDS through S/P. Next, the signal is converted back to the frequency domain through DFT and recovered through P/S [20]. In the channel, the transmitted signal is multiplied by the channel response and added with noise to form the received signal. After the above processing, the received signal is input into the DNN model along with the transmission symbol for offline training. The DNN model learns the relationship between transmitted symbols and received signals to predict and restore the original transmitted symbols. Through online deployment, the trained DNN model can be utilized to achieve real-time signal detection.

### 2.2. CSD optimization model integrating multi-DNN algorithm

**Fig 4:**
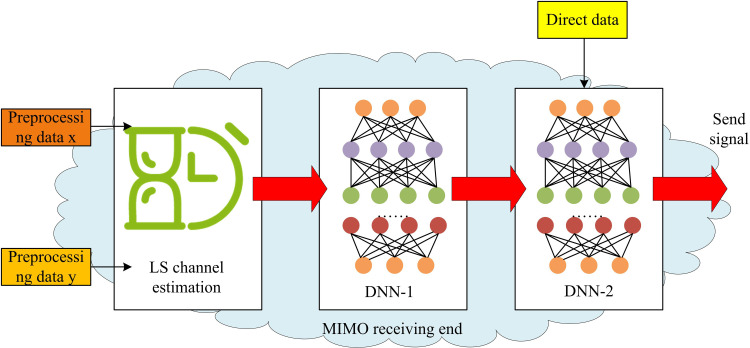
CSD optimization with Multi-DNN fusion.

Although data-driven signal detection models based on DNN can detect communication signals. DNN models have high complexity. MIMO-OFDM systems also have high complexity. Therefore, the combination of DNN and MIMO-OFDM may further increase the complexity of the system [21]. In addition, the performance of DNN models largely depends on the quality and quantity of training data. However, in communication systems, the real communication environment is often complex and unpredictable, making it difficult to fully simulate. Therefore, obtaining high-quality training data is often very challenging. Furthermore, for MIMO-OFDM, synchronization and channel estimation are key components of the system. However, DNN may face challenges in rapidly changing communication environments, leading to synchronization bias and channel estimation bias, ultimately resulting in decreased signal detection performance. Given these issues, the study uses Multi-DNN to optimize the MIMO-OFDM model. The difference between Multi-DNN and DNN is that Multi-DNN is based on the idea of ensemble learning, which trains multiple DNN models and fuses them to obtain more accurate detection results. In the original MIMO-OFDM detection model, Multi-DNN is mainly used for optimizing MIMO, as shown in Fig 4.

In Fig 4, the data received by the receiving end are divided into two groups, one is direct data and the other is preprocessed data y and x. After the preprocessed data are subjected to channel estimation using the least squares method, it is sequentially processed using dual DNNs [22]. The last layer of DNN utilizes the channel information output by the previous layer of DNN and the directly received data to perform signal detection tasks, ultimately outputting an estimated transmission signal. Among them, the channel information output by the DNN of the first layer is shown in formula (5).


$$DNN_{1}({\tilde{H}}_{LS})=W^{0}\phi^{L}(W^{L}\phi^{L-1}(\cdots \phi^{1}(W^{1}{\tilde{H}}_{LS}+a^{1})\cdots )+a^{L})+a^{0}$$ (5)

**Fig 5:**
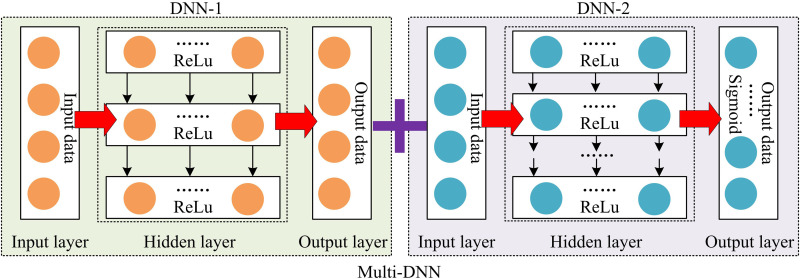
Internal architecture of Multi-DNN.

In formula (5), $DNN_{1}({\tilde{H}}_{LS})$ is the channel information output by the DNN of the first layer. W and ϕ are the weight matrix and activation function of the corresponding layer. ${\tilde{H}}_{LS}$ is the initial channel estimation matrix based on the least squares method. a is the bias vector of the corresponding layer, used to adjust the output of the activation function. The introduction of Multi-DNN in the research model relies on the combination of dual DNNs. Each layer of DNN can be regarded as an independent predictor that can learn from different perspectives or feature spaces on the input data. Therefore, to fully utilize the complementarity of Multi-DNNs and improve the overall detection accuracy, this study uses ReLU in the hidden layers of the first and second DNNs to address the gradient explosion problem. Sigmoid is used in the output layer of the second DNN to simplify the decision-making process [23]. Fig 5 shows the structure of Multi-DNN.

In Fig 5, the introduced Multi-DNN is composed of two DNNs, each with an internal structure of “input layer hidden layer output layer”. The loss functions of the two DNNs are shown in formula (6).


$$\left\{ \begin{array}{c} L_{1}=\frac{1}{\left|\Omega \right|}\sum_{H\in \Omega }{\left\|\tilde{H}-H\right\|}^{2}\\ L_{2}=\frac{\sum_{i=1}^{N}{\left({\tilde{m}}_{i}-m_{i}\right)}^{2}}{N} \end{array}\right.$$ (6)

**Fig 6:**
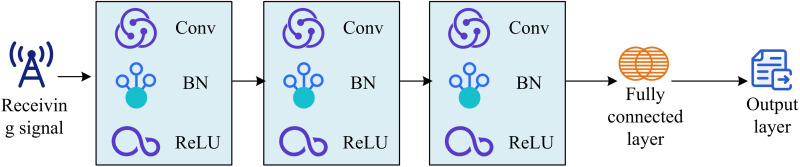
Internal structure of DCNet.

In formula (6), $L_{1}$ and $L_{2}$ are the loss functions of the first and second DNNs. Ω is the dataset used for training the corresponding DNN. $\tilde{H}$ and H are the estimated and true channel matrix output by the first DNN. N is the gross of samples in signal detection. ${\tilde{m}}_{i}$ is the estimated transmission symbol output by the second DNN. $m_{i}$ is the real transmission symbol. Multi-DNN is mainly utilized to improve the MIMO part of MIMO-OFDM models, without optimizing the OFDM part, which is sensitive to frequency offset due to the use of a large number of subcarriers [24]. Frequency deviation can cause interference between subcarriers, thereby affecting the overall performance of the detection model [25]. Based on this, this study proposes to further introduce a DCNet to optimize the OFDM part of the MIMO-OFDM model. The internal structure of DCNet is exhibited in Fig 6.

In Fig 6, the received signal input undergoes a series of Convolutional Layers (ConvL) for processing. Each ConvL is followed by a Batch Normalization (BN) layer and a ReLU [26]. This structure not only enables the extraction of signal features but also enables the network to learn complex feature representations simultaneously. After being processed through multiple layers of convolution and activation layers, the signal will be input into a Fully Connected Layer (FCL). The FCL is responsible for mapping the extracted features to the output layer and ultimately generating a decoded signal [27–28]. The entire network structure is optimized end-to-end by minimizing the difference between the output signal and the original transmission signal, effectively reducing inter sub-carrier interference and improving the overall performance [29–30]. The loss function used by DCNet is shown in formula (7).


$$L_{DCNet}(\tilde{y},y)=\sum_{i=1}^{k}{\left[{\tilde{y}}_{i}-y_{i}\right]}^{2}$$ (7)

**Fig 7:**
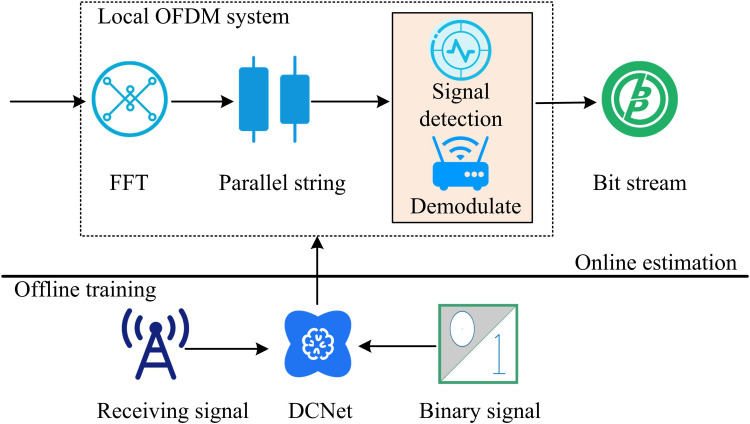
Optimization process of DCNet for OFDM.

In formula (7), $L_{DCNet}$ is the loss function of DCNet, utilized to measure the difference between the model’s predicted output and the true label. k is the overall samples. ${\tilde{y}}_{i}$ denotes the i-th output value predicted by the model. $y_{i}$ is the actual i-th label value. The specific process of optimizing OFDM based on DCNet is shown in Fig 7.

In Fig 7, the working scope of DCNet is similar to that of Multi-DNN, both of which belong to offline training [31]. DCNet processes binary signals and received signals before transmitting them to the online estimation of OFDM for optimization. Among them, the preprocessing step is to first normalize the received signal, limiting its amplitude range to the [-1,^1^] interval. For OFDM signals, FFT is performed after removing the CP, and the FDS is grouped by subcarriers as DCNet input. MIMO signals are estimated through least squares channel estimation. The real and imaginary parts are separated into two channels and input into Multi-DNN.

It is not difficult to find that in model training, the research adopts a phased collaborative optimization strategy, which independently trains and jointly fine-tunes the Multi-DNN and DCNet modules. The Multi-DNN module optimizes the channel estimation and signal detection of MIMO systems in stages through a cascaded network structure. The first layer of DNN takes the channel matrix estimated by least squares as input and outputs robust channel estimation results through the nonlinear transformation of the ReLU activation function. The second layer of DNN is based on the channel information output from the first layer and the original received signal, utilizing the threshold characteristics of the Sigmoid activation function to complete the symbol decision. During the training process, two layers of DNNs are independently optimized using Mean Square Error (MSE) and cross-entropy loss functions. The network parameters are gradually adjusted through a gradient descent algorithm to ensure that each layer of the network can efficiently learn its specific task feature representation. The DCNet module focuses on solving the problem of inter-subcarrier interference in OFDM systems. Its training takes the frequency domain representation of the received signal as input, extracts local interference patterns between subcarriers through a multi-layer convolutional structure, and combines BN layers to stabilize gradient propagation. After each convolution layer, a ReLU activation function is applied to enhance the nonlinear expression ability. Finally, the decoded signal is output through a FCL. The training objective is to minimize the MSE between the decoded signal and the true label. The Adam optimizer is used to dynamically adjust the learning rate, ensuring that the model can effectively capture the interference features in high-frequency signals. After completing independent pre-training, Multi-DNN and DCNet are integrated into the complete MIMO-OFDM system for end-to-end joint fine-tuning. During the joint training phase, a dynamic weighting strategy is used to balance the loss functions of the two sub-networks, ensuring the coordinated optimization of channel estimation, signal detection, and subcarrier interference suppression. To avoid the problem of gradient explosion, a gradient pruning technique is adopted to constrain the parameter update amplitude, while a cosine annealing learning rate scheduling strategy is introduced to improve the convergence stability of the model. Finally, by combining phased training with joint fine-tuning, the system level performance is improved while retaining the professional capabilities of each module, demonstrating excellent overall performance on the test set. Based on the above, the improvement of MIMO-OFDM model can be achieved by introducing Multi-DNN and DCNet.

## 3. Results

### 3.1. Performance verification based on CSD Model

**Fig 8:**
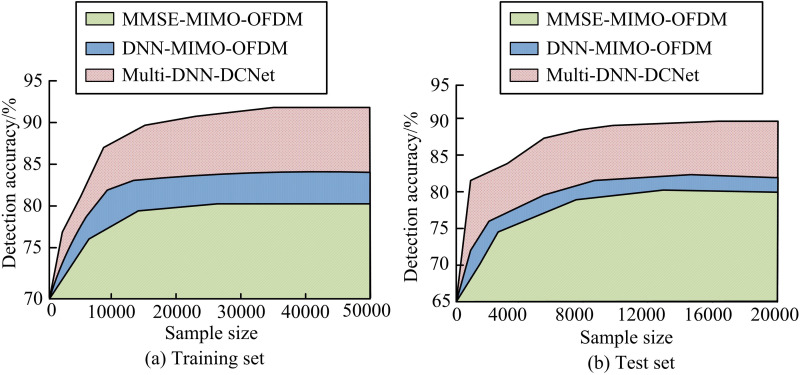
Detection accuracy of each model on the training and testing sets.

To validate the performance of the research model in CSD, a simulation platform is built using Python and TensorFlow framework. The hardware configuration is Intel i7-12700K + RTX 3080 Ti. The Nakagami-m fading channel model is used in the simulation, which can flexibly simulate various wireless communication environments from mild to severe fading. The channel matrix H is time-varying, and the channel state of each time slot is independently generated, which can simulate the dynamic changes in actual communication. The experiment uses Adam optimizer and dynamically adjusts using cosine annealing strategy, with an initial learning rate set to 0.001. The batch size used for neural network training is 128, with a cross entropy loss function, a training period of 200 epochs, and an early stopping patience value of 10 epochs. The simulation data are generated using the Nakagami-m fading channel model, with a parameter of m = 2.5, to simulate a typical urban multi-path environment. The training set contains 100,000 samples and the test set contains 20,000 samples, each containing 85,000 signal points. The sampling rate is set to 50 MHz (2.4GHz band) and 90 MHz (5GHz band) based on device parameters. The experiment adopts a comparative method, incorporating the traditional MMSE-based detection model (MMSE-MIMO-OFDM) and the DNN-based data-driven model (DNN-MIMO-OFDM) as comparison. The research model is named as “Multi-DNN-DCNet”. The experiment first tests the detection accuracy of each model on the training and testing sets, as shown in Fig 8.

**Fig 9:**
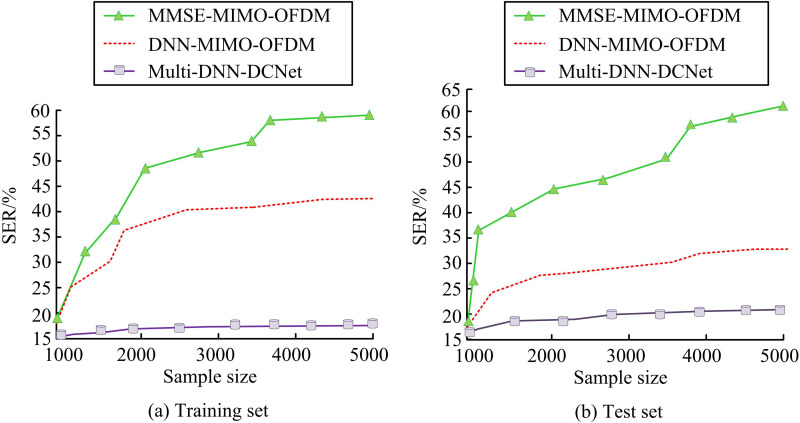
SER of each model on the dataset.

Fig 8(a) shows the detection accuracy of each model on the training set. The figure shows that the accuracy of the MMSE-MIMO-OFDM model is the lowest, at 80.1%. The accuracy of the DNN-MIMO-OFDM model is 84.3%, significantly better than MMSE-MIMO-OFDM (*p* < 0.01). The accuracy of the Multi-DNN-DCNet model is 93.8%, significantly higher than the first two (*p* < 0.001). Figure 8(b) shows the detection accuracy of each model on the test set. Consistent with the training set, the MMSE-MIMO-OFDM model has the lowest accuracy, specifically 78.6%, while the DNN-MIMO-OFDM model has 82.8%. In comparison, the DNN-MIMO-OFDM model still maintains a significant advantage (*p* < 0.05). However, compared with DNN-MIMO-OFDM, the Multi-DNN-DCNet model demonstrates the strongest generalization performance with an accuracy of 90.7% (*p* < 0.01). In addition, the experiment also verifies the SER of each model, as shown in Fig 9.

**Fig 10:**
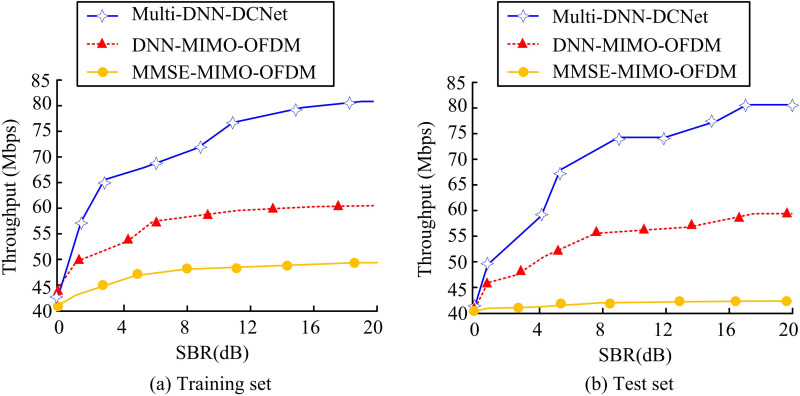
Throughput of each model on the two sets.

Fig 9 (a) shows the error rate results of each model on the training set. The figure shows that the MMSE-MIMO-OFDM model has the highest BER of 59.2%, while the DNN-MIMO-OFDM model has a BER of 42.2%, significantly lower than MMSE-MIMO-OFDM (*p* < 0.001). In contrast, the Multi-DNN-DCNet model has the lowest BER, specifically 17.6%. Compared with the DNN-MIMO-OFDM model, its performance is better (*p* < 0.001). Fig 9 (b) shows the BER results of each model on the test set. Consistent with the performance of the training set, the MMSE-MIMO-OFDM model maintains the highest BER of 60.2%. The error rate of the DNN-MIMO-OFDM model reaches 31.4%, and compared with the MMSE-MIMO-OFDM model, DNN-MIMO-OFDM still has significant advantages (*p* < 0.01). However, compared with DNN-MIMO-OFDM, the Multi-DNN-DCNet model exhibits the best robustness with a minimum error rate of 18.1% (*p* < 0.005). As the BER directly reflects the number of transmission errors, the above statistical results further confirm that the Multi DNN DCNet model has significant advantages in error control.. Furthermore, the experiment also examines the throughput of each model, as shown in Fig 10.

**Fig 11:**
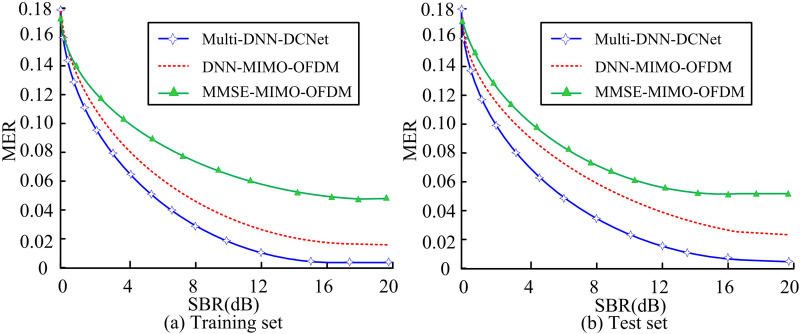
Throughput of each model on the two sets.

Fig 10 (a) shows the throughput of each model on the training set. From the graph, the throughput of the MMSE-MIMO-OFDM model is the lowest, at 49.4%, while the throughput of the DNN-MIMO-OFDM model is 60.7%, significantly better than the MMSE-MIMO-OFDM model (*p* < 0.01). However, compared to DNN-MIMO-OFDM, the Multi-DNN-DCNet model achieves a statistically significant improvement (*p* < 0.001) with a throughput of 81.3%. Fig 10 (b) shows the throughput performance of each model on the test set. The MMSE-MIMO-OFDM model maintains the lowest throughput (41.2%), while the DNN-MIMO-OFDM model (59.4%) remains significantly higher than the MMSE-MIMO-OFDM model (*p* < 0.05). Compared with the DNN-MIMO-OFDM model, the Multi-DNN-DCNet model has the highest throughput of 81.2% and the best performance (*p* < 0.001). In CSD, high throughput directly reflects signal processing efficiency. The above statistical differences fully demonstrate the superiority of the Multi-DNN-DCNet model in real-time performance. In addition, the experiment also tests the Modulation Error Ratio (MER) of the model under different SNRs, as shown in Fig 11.

Fig 11 (a) shows that the MER of the MMSE-MIMO-OFDM model is the highest at 0.056, while that of the DNN-MIMO-OFDM model is 0.018, significantly lower than the baseline model (*p* < 0.001). However, in comparison, the Multi-DNN-DCNet model has a lower error rate of 0.004 (*p* < 0.001). Fig 11 (b) shows the performance of each model on the test set, and similarly to the training set, both exhibit the same trend. MMSE-MIMO-OFDM maintains the highest error rate (0.057). DNN-MIMO-OFDM (0.032) still significantly outperforms the benchmark (*p* < 0.05). Multi-DNN-DCNet demonstrates the strongest signal fidelity ability with an error rate of 0.006 (*p* < 0.001 compared to the sub-optimal model). Due to the fact that MER directly reflects signal quality, these statistical results confirm that Multi-DNN-DCNet has significant advantages in improving communication efficiency (Cohen’s d = 1.82).

### 3.2. Case analysis based on CSD Model

The above study only tested the comprehensive performance of the research model in communication detection. To further explore the practical application effect of the model, this study conducted a case analysis. Considering that WiFi datasets typically contain a large amount of communication signal data, the experiment used WiFi datasets as experimental data. The collection sites for this WiFi dataset were all completed in a university laboratory, with Line of Sight (LOS) propagation as the main collection environment. Table 1 shows the specific equipment parameters for signal acquisition.

**Table 1:** Signal acquisition equipment and its parameters.

Signal acquisition equipment	FSQ26	FSW26	FSW26	FSV13
Number of modules	12	110	110	300
Channel number	1	1	6	1
Sampling rate	50M	50M	90M	50M
Number of samples	45	210	110	110
Number of signal points	85000	45000	85000	45000

**Fig 12:**
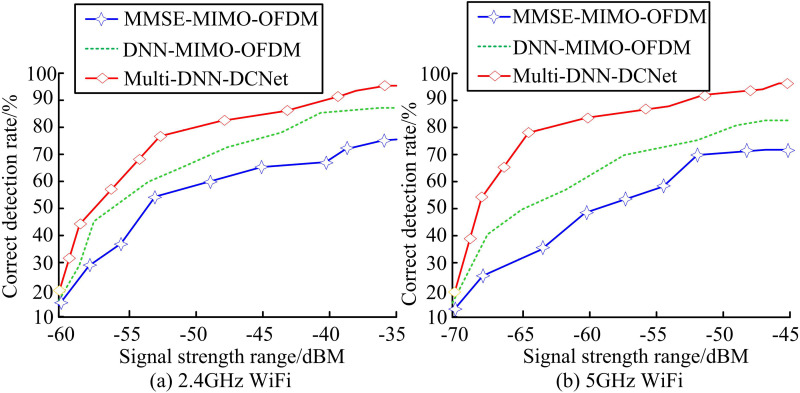
Detection accuracy of various models in different signal types.

In Table 1, for WiFi module devices, the experiment includes two types: 2.4G WiFi module and 5G WiFi module, using ESP8266 and RM28E device models. The experiment first tests the detection accuracy of the model in different signal types, as shown in Fig 12.

**Fig 13:**
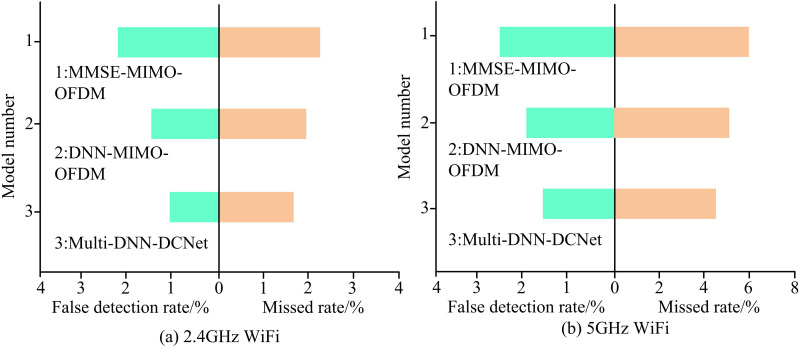
FDR and MDR of the model in two signal types.

Fig 12 (a) shows that in 2.4GHz WiFi signals, the MMSE-MIMO-OFDM model has the lowest detection accuracy (72.1%), while the DNN-MIMO-OFDM model (81.3%) is significantly better than the baseline model (*p* < 0.01). However, compared with the Multi-DNN-DCNet model, this model achieves an accuracy of 91.5% and performs better (*p* < 0.001). The test results of 5GHz WiFi signal in Fig 12 (b) show the same trend. The MMSE-MIMO-OFDM model achieves the lowest accuracy rate (69.7%), while the DNN-MIMO-OFDM model (80.3%) remains significantly higher than the benchmark (*p* < 0.05). The Multi-DNN-DCNet model achieves an accuracy rate of 91.6%, significantly better than the sub-optimal model (*p* < 0.001). The experiment also compares the False Detection Rate (FDR) and Missed Detection Rate (MDR) of the model in different signal types, as shown in Fig 13.

Fig 13(a) shows that in 2.4GHz WiFi signals, the MMSE-MIMO-OFDM model has the highest FDR (2.4%) and MDR (2.1%), while the DNN-MIMO-OFDM model (1.5% FDR, 1.8% MDR) shows significant improvement compared to the baseline model (*p* < 0.05). The Multi-DNN-DCNet model achieves the lowest error rate (1.9% FDR, 1.6% MDR), with a particularly significant improvement in MDR (*p* < 0.01). In the 5GHz WiFi signal shown in Fig 13(b), the MMSE-MIMO-OFDM model still maintains the highest error rate (2.7% FDR, 5.7% FDR), while the DNN-MIMO-OFDM model (1.9% FDR, 4.8% FDR) has significantly lower FDR than the baseline (*p* < 0.05). The Multi-DNN-DCNet model shows statistically significant advantages in both types of error rate indicators with an FDR of 1.5% and 4.2% (*p* < 0.01). The real-time performance of the model is crucial for signal detection in practical applications. Moreover, due to the superior performance of the research model compared to the comparative models analyzed above, this study evaluates the resource consumption of the model at different times based on this case. Table 2 presents detailed results.

**Table 2:** Evaluation of resource consumption of the model.

Point of time	Evaluation indicators	Evaluation results
T1	Detection speed = 120signals/s	Faster
	CPU usage = 24%	Lower
	Memory footprint = 180MB	Moderate
	Average delay = 50ms	Low latency
	Model loading time = 2.4s	Faster
T2	Detection speed = 121signals/s	Faster
	CPU usage = 25%	Lower
	Memory footprint = 178MB	Moderate
	Average delay = 51ms	Low latency
	Model loading time = 2.3s	Faster
T3	Detection speed = 120signals/s	Faster
	CPU usage = 25%	Lower
	Memory footprint = 178MB	Moderate
	Average delay = 52ms	Low latency
	Model loading time = 2.5s	Faster

Table 2 lists the resource consumption evaluation results of the proposed model at different time points. This study randomly selects three time points, among which at time T1, the detection speed of the model is 120 signals/s, the CPU usage is 24%, the memory usage is 180 MB, the average latency is 50 ms, and the loading time of the model is 2.4 s. At time T2 and T3, the detection speed of the model is 121signals/s and 120signals/s, the CPU usage is 25%, the memory usage is 178MB, the average latency is 51ms and 52ms, and the loading time of the model is 2.3s and 2.5s. At three different time points, the research model is evaluated to have fast detection speed and low average delay in WiFi signal detection, thereby meeting real-time requirements. In addition, the low CPU usage indicates that the model does not have high requirements for computing resources. Moderate memory usage indicates that it can run on most devices. Moreover, the loading time of the models has also been evaluated as relatively fast, making them suitable for scenarios that require quick startup. Overall, the proposed model can be well applied to the detection of communication signals.

## 4. Discussion and conclusion

To improve communication quality and ensure information security, this study proposed a MIMO-OFDM system based on the combination of MIMO and OFDM technologies, and introduced the Multi-DNN algorithm to optimize the MIMO part on this basis. It adopted the DCNet algorithm to optimize the OFDM part and constructed a Multi-DNN-DCNet. The paper first compared the Multi-DNN-DCNet’s performance with MMSE-MIMO-OFDM and DNN-MIMO-OFDM models. The results demonstrated that the accuracy of Multi-DNN-DCNet on the simulation training set was 93.8%, SER was 17.6%, throughput was 81.3%, and MER was 0.004. Its accuracy on the simulation test set was 90.7%, SER as 18.1%, throughput was 81.2%, and MER was 0.006. Compared with other models, Multi-DNN-DCNet performed the best in accuracy, throughput, SER, and MER metrics. In the high-frequency communication scenario of OFDM systems, Multi-DNN-DCNet outperforms other models (such as MMSE-MIMO-OFDM, DNN-MIMO-OFDM, etc.) due to its dual layer network structure achieving collaborative gain through phased optimization. The first layer of DNN generates a robust channel matrix based on least squares initial estimation. The second layer of DNN utilizes the threshold characteristics of the Sigmoid activation function to achieve symbol level accurate decision-making. At the same time, the DCNet module effectively captures the interference patterns between subcarriers through the local receptive field characteristics of the ConvL. Combined with the BN layer, it significantly improves the gradient stability under high-frequency signals. This joint optimization mechanism enables the system to exhibit better BER and throughput performance in both simulation and measurement scenarios. This result is consistent with the research findings of Xie H et al. The multimodal data transmission system proposed by them exhibits stronger robustness in the low SNR range [32]. This indicates that the data-driven optimization method incorporating Multi-DNN can significantly improve the performance of CSD.

To further verify the practical application effect, this study collected two types of WiFi signals, 2.4 GHz and 5 GHz, for experiments. The detection accuracy of the Multi-DNN-DCNet model in 2.4 GHz WiFi signals reached 91.5%, with FDR and MDR of 1.9% and 1.6%, respectively. Its detection accuracy in 5 GHz WIFI signals was 91.6%, with FDR and MDR of 1.5% and 4.2%. In the 2.4 GHz frequency band, Multi-DNN effectively suppressed co-frequency interference in dense channel environments through a double check mechanism. In the 5 GHz high-frequency band, DCNet structure adapted to greater frequency selective fading through multi-scale feature fusion, and its MDR performance was improved by about 40% compared to traditional methods. These indicators were significantly better than the MMSE-MIMO-OFDM and DNN-MIMO-OFDM models. In addition, in the resource consumption assessment, the Multi-DNN-DCNet model achieved a detection speed of 120 signals/s at time T1, an average latency of 50 ms, a model loading time of only 2.4 s, a CPU usage rate as low as 24%, and a memory usage of 180 MB. Compared with some existing real-time CSD systems, such as Sabat D et al.‘s research [33], the Multi-DNN-DCNet model performed well in terms of CPU and memory usage. In the other two randomly selected moments, its value was also relatively stable, close to the T1 moment. This indicates that the model can meet real-time requirements, is suitable for most devices, and has low computational resource requirements, making it suitable for fast startup scenarios. The results of this study are similar to those of Tong X et al. The proposed joint scheme of multi-user communication and environmental perception achieves efficient sensor communication integration by utilizing the sparsity of user signals and environment [34]. However, compared with Tong X et al.’s method, the Multi-DNN-DCNet model performs better in terms of accuracy and resource efficiency in signal detection, especially demonstrating stronger adaptability in real-time requirements in complex communication environments.

Although the Multi-DNN-DCNet model performs well in CSD, there are still certain limitations. For example, the study only conducted experiments on WIFI signals and did not involve other types of communication signals such as Bluetooth, Zigbee, LTE, 5G, etc. This scenario limitation may affect the generalization ability of the model in heterogeneous communication networks, such as IoT hybrid network environments. Especially when facing differentiated signal characteristics such as frequency hopping spread spectrum (Bluetooth) and low-power narrowband (Zigbee), it may be necessary to re optimize existing parameter configurations. In addition, the model validation is completed in a laboratory LOS environment. In actual deployment, multi-obstacle Non-Line of Sight (NLOS) scenarios may weaken the suppression effect of DCNet on multi-path effects, and it needs to be enhanced through a channel adaptation module. Although the model has a low dependence on computing resources during the optimization process, its performance on extremely low-power devices such as energy harvesting sensor nodes still needs further validation. Future research can expand multi-protocol support capabilities by introducing lightweight algorithms with adaptive signal features. Second, future research can develop FPGA-based hardware acceleration architecture to adapt to resource constrained devices. Third, it can supplement NLOS scenario testing data in complex physical environments.

In summary, the proposed Multi-DNN-DCNet model significantly improves the performance of CSD by combining MIMO-OFDM system, Multi-DNN algorithm, and DCNet algorithm. Experimental results have shown that the model performs well in both simulated datasets and actual WIFI signal detection, with high real-time performance and low computational resource requirements, making it well suited for CSD. Based on the limitations of current research, future work can focus on breaking through in the following directions: firstly, to address the problem of the insufficient generalization ability of the model in communication protocols other than WiFi (such as Bluetooth and Zigbee), a cross-protocol joint training framework needs to be constructed. By designing a hybrid training mechanism that can adapt to frequency hopping spread spectrum and narrowband signal features, the model can dynamically recognize and process differentiated signal patterns in heterogeneous networks. Secondly, to enhance the practicality of the model in complex NLOS environments, an online learning module that integrates real-time channel state feedback should be developed. Moreover, it can be combined with reinforcement learning to dynamically optimize the multi-path suppression strategy of DCNet. At the same time, an open test dataset containing multiple obstacle scenarios should be established to verify the actual deployment effect. Finally, it is necessary to explore a model lightweight scheme based on neural architecture search for the application requirements of extremely low-power devices. This is done to ensure detection accuracy while reducing resource consumption to an acceptable range for energy-harvesting sensor nodes through joint optimization of hardware perception, such as FPGA bit width adaptive compression. These improvements will systematically address the key bottlenecks in the process of transitioning existing models from laboratory environments to real scenarios.
